# New magneto-polaron resonances in a monolayer of a transition metal dichalcogenide

**DOI:** 10.1038/s41598-023-27404-x

**Published:** 2023-01-06

**Authors:** Carlos Trallero-Giner, Darío G. Santiago-Pérez, Vladimir M. Fomin

**Affiliations:** 1 Institute for Integrative Nanosciences (IIN), Leibniz Institute for Solid State and Materials Research (IFW) Dresden, Helmholtzstraße 20, D-01069 Dresden, Germany; 2 Universidad Autónoma del Estado de Morelos, Ave. Universidad 1001, CP 62209 Cuernavaca, Morelos, México; 3 Laboratory of Physics and Engineering of Nanomaterials, Department of Theoretical Physics, Moldova State University, str. A. Mateevici 60, MD-2009 Chişinău, Republic of Moldova

**Keywords:** Surfaces, interfaces and thin films, Electronic properties and materials, Quantum mechanics

## Abstract

Transition metal dichalcogenide (TMD) semiconductors are two-dimensional materials with great potential for the future of nano-optics and nano-optoelectronics as well as the rich and exciting development of basic research. The influence of an external magnetic field on a TMD monolayer raises a new question: to unveil the behavior of the magneto-polaron resonances (MPRs) associated with the phonon symmetry inherent in the system. It is shown that the renormalized Landau energy levels are modified by the interplay of the long-range Pekar–Fröhlich (PF) and short-range deformation potential (DP) interactions. This leads to a new series of MPRs involving the optical phonons at the center of the Brillouin zone. The coupling of the two Landau levels with the LO and $A_1$ optical phonon modes provokes resonant splittings of double avoided-crossing levels giving rise to three excitation branches. This effect appears as bigger energy gaps at the anticrossing points in the renormalized Landau levels. To explore the interplay between the MPRs, the electron-phonon interactions (PF and DP) and the couplings between adjacent Landau levels, a full Green’s function treatment for the evaluation of the energy and its life-time broadening is developed. A generalization of the two-level approach is performed for the description of the new MPR branches. The obtained results are a guideline for the magneto-optical experiments in TMDs, where three MPR peaks should be observable.

## Introduction

Magneto-polaron (MP) resonances have been extensively investigated in three-dimensional (3D) semiconductors^1–4^. When the energy of a higher Landau level *N* is perturbed by another Landau level *M* of a lower energy accompanied by an optical phonon, it happens that for a certain value of the magnetic field there will be an intersection between the two quantum states *N*, *M*. For this magnetic field these states are degenerate. A resonant MP coupling occurs, when the energies of two Landau levels, $E_N$ and $E_M$, are separated by the energy of one optical phonon, $E_N-E_M=\hbar \omega _0$ at $N>M$. These two states are resonantly coupled due to the electron-phonon interaction (EPI), and each Landau level splits into two branches. This effect is known to be particularly important for cyclotron-resonance experiments^5^, magneto-optical properties^6^ and magneto-Raman scattering^7–9^. Understanding and design of the polaron phenomena in nanostructures is one of the key problems of modern nanophysics (see, e.g., Ref.^10^ and references therein). Special attention has been paid to the control over the polaron energy and other polaron properties in response to the concerted actions of an external magnetic field *B* and a geometric confinement^11,12^, such as in quantum wires^11^ and quantum wells^12^. The emergence of a new family of two-dimensional (2D) transition metal dichalcogenides (TMDs) has revealed their extraordinary potential applications for various technologies^13,14^, as well as for fundamental research in spin-liquid physics based on a twisted bilayer of TMDs^15^, where a correlated insulating phase has been reported^16,17^. TMDs with a direct band gap in the visible and near-infrared regions^18^ are excellent candidates for next-generation nanoelectronics^19^ and have greatly promoted basic research. One of the most exciting effects in TMDs is the existence of charge density waves^20^, opening a new avenue to study the phenomena of strongly correlated systems as Mott phases^21^, magnetic order^22^, and superconductivity^23^ with perspective for applications in nanoelectronic devices^24^.

The EPI in 2D TMD is a fundamental tool for understanding transport properties^25^, hot luminescence^26^, interband and intraband relaxation processes^27^, phonon-assisted photoluminescence^28^, gap-renormalization effect^29^, dielectric properties^30^, Raman spectroscopy^31,32^, and also many of the aforementioned correlation phenomena. Huge first-order magneto-Raman intensities are observed in a monolayer (ML) and a bilayer of MoS$_2$^33^. The impact on the magneto-optical absorption in TMD MLs under extremely high *B* up to 91 T is revealed in Ref.^34^. Employing the magneto-spectroscopic investigations, the authors determine essential materials parameters, such as exciton masses, exciton binding energies, exciton radii, and dielectric properties in ML of MoS$_2$, MoSe$_2$, MoTe$_2$, and WS$_2$. The MP processes, due to the electron-LO Pekar–Fröhlich (PF) interaction, have been reported for ML of MoS$_2$ and WS$_2$^35^. The behavior of the exciton-polaron magnetic interaction with correlated electronic states in a moiré WSe$_2$ superlattice has been recently unveiled^36^. In particular, the magnetic field enables the observation of attractive and repulsive exciton-polarons due to the exchange interactions with the moiré pinned charge carriers. Recently, the dynamics of the MP condensate and the effect of the inter-valley MP resonance on the cyclotron energy in TMD materials have been addressed in Refs.^37^ and^38^, respectively.

**Figure 1:**
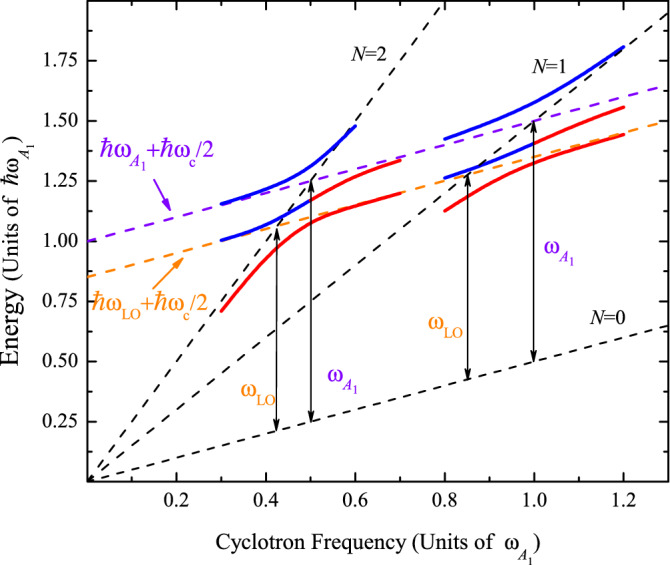
Scheme of the electronic excitations in ML TMD as a function of the cyclotron frequency, $\omega _c$, in the range of *B* where the energies are in resonance with the $\omega _{\text {LO}}$ and $\omega _{A_1}$ phonons due to the EPI. Blue (red) solid lines represent the lower and upper branches above (below) the threshold $\omega _{\text {LO}}$ and $\omega _{A_1}$. The evolution and the interplay between the PF and $A_1$-DP contributions to the two resonant anticrossing magneto-energies are shown as a function of *B* (see text for details). Black dashed lines indicate the bare Landau levels $\hbar \omega _c(N+1/2)$ for $N=0$ to 2 and the orange (violet) dashed lines correspond to the bare excited states $\hbar \omega _{\text {LO}} + \hbar \omega _c/2$ ($\hbar \omega _{A_1} + \hbar \omega _c/2$).

In ML of TMDs, optical phonons with the irreducible representations $A_1$ and $E^{'}(\text {LO})$ at the center of the Brillouin zone (BZ)^39^ couple electronic states via intravalley EP mechanisms at K or K$^{'}$-valleys^40^. Consequently, the interaction of electrons with the short-range A$_1$-homopolar deformation potential (DP) and the long-range PF contributions must be considered for a correct evaluation of the polaron properties^41^. The peculiarity of the TMD with two independent electronic intravalley transitions assisted by optical phonons with different symmetry introduces new qualitative and quantitative differences from the well-known magneto-phonon resonances in semiconductors. In this case, we expect dissimilar magneto-phonon resonance effects when two electronic Landau levels around the K and K$^{'}$ points are coupled with the $A_1$ or $E^{'}(\text {LO})$-modes. Hence, for certain values of the magnetic field ${ B}_{A_1}$ and ${ B}_{\text {LO}}$, the phonon energies $\hbar \omega _{\text {A}_1}$ or $\hbar \omega _{\text {LO}}$ can match two Landau levels, and, under this circumstance, we have three excitation branches. To get a qualitative view of the main physical characteristics of the MP process in TMDs, in Fig. 1 we illustrate the evolution of the electronic excitations in the magnetic fields close to the resonances with the optical phonons of energy $\hbar \omega _{\text {LO}}$ and $\hbar \omega _{A_1}$. Two degeneracies occur between each Landau level with the quantum number *N* (black dashed lines) and two excited sates (orange and violet). Two crossing points at ${ B}_{A_1}$ and ${ B}_{\text {LO}}$ are lifted by two different kinds of the EPI. By the inherent symmetry of each phonon mode, we observe that with increasing magnetic field the energy branch above the phonon energy $\hbar \omega _{\text {LO}}+\hbar \omega _{c}/2$ (orange dashed lines) evolves into the resonance with the lowest Landau level below the excited state $\hbar \omega _{A_1}+\hbar \omega _{c}/2$ (violet dashed lines).

The present work unveils the MP effects on the electronic levels in ML of MX$_2$ (M = Mo, W and X = S, Se)^42^ in the presence of a static *B* perpendicular to the plane. Our work relies on a rigorous description of MP resonances on the basis of the Green’s function formalism with due account for the role played by two optical phonons, $A_1$-homopolar and longitudinal optical $E^{'}(\text {LO})$-modes. In addition, we validate the relative contribution of the PF and the DP couplings to the MP energy. The broadening effects on the MP states play an important role in the evaluation of the magneto-optical properties, in particular, magneto-Raman scattering^8,43^. Likewise, the knowledge of the electronic life-time and its dependence on the applied magnetic field is of essential importance for a correct examination of many aforementioned processes. Moreover, the very feasibility of the observation of the anticrossing in the MP spectrum depends on the width of the electronic levels, especially, for highly excited states.

In the present formalism, for the evaluation of the self-energy operator, we include the coupling between all Landau states: the electron-optical phonon interactions couple each Landau level *N* with all others. By solving the Dyson equations, we obtain the dependence of the renormalized energy and the life-time broadening on *B* for the above-described family of MX$_2$.

## Results and discussions

The coupling of the Landau levels owing to the EPI breaks the symmetry of an electronic gas in an applied external *B* leading to the occurrence of new quantum numbers *n* in place of *N*. The solution of the Dysion equation for the full Green’s function provides a set of levels enumerated in the order of increasing energy (see Sect. **General Formulation**).

### Magneto-polaron energy vs. magnetic field

For a given ${ B}$, the index *n* denotes the polaron ground state energy plus two infinite sets of excited states with the unperturbed energies $\epsilon _{N}=\hbar \omega _{c}(N+1/2)$ and the relevant asymptotes $\hbar \omega _{\text {LO}}+(p_1+1/2)\hbar \omega _c$, $\hbar \omega _ {A_1}+(p_2+1/2)\hbar \omega _c$ $(p_1, p_2=0,1,2,...$). In the present case, we obtain two series of anticrossings at two different cyclotron frequencies

1 $$\begin{aligned} \omega _c^{(\text {LO})}(B_{\text {LO}})=\frac{\omega _{\text {LO}}}{N-p_1}\; ; \;\;\; \;\;\; \omega _c^ {(A_1)}(B_{A_1})=\frac{\omega _{A_1}}{N-p_2}\;. \end{aligned}$$

The crossing points (at the magnetic fields ${ B}_{\text {LO}}$ and ${ B}_{A_1}$) between the Landau level *N* and the adjacent excited states with phonon frequencies $\omega _{\text {LO}}$ and $\omega _{A_1}$ are responsible for the existence of a three-level anticrossing. The value *B*, where resonances for the MX$_2$ family occur, ranges from 16 T to 30 T, the lowest value for the WSe$_2$ and the highest for MoS$_2$. One of our key results is the solution of the eigenvalue-problem (see Eqs. (6) and (7) in the Sect. **General Formulation)** including two anticrossing series of Eq. (1).

**Figure 2:**
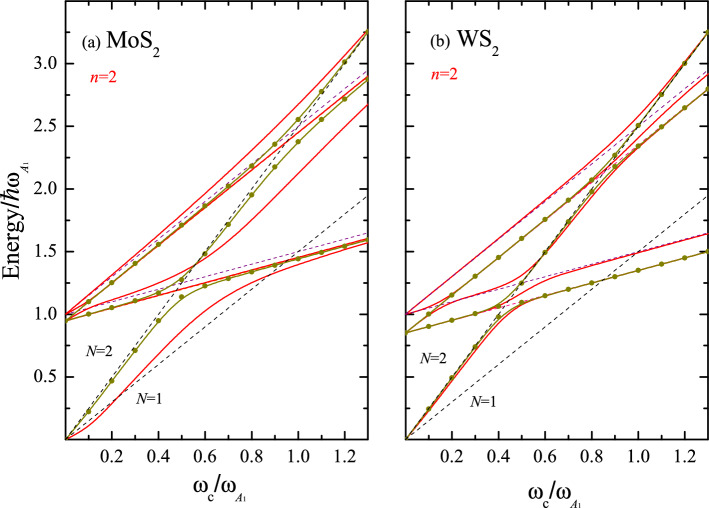
The total MP energies, ${\hat{\epsilon }}_n/\hbar \omega _{A_1}$, for $n=2$ (red lines) vs the reduced cyclotron frequency $\omega _c/\omega _{A_1}$ for (**a**) MoS$_2$ and (**b**) WS$_2$. The contribution of the PF interaction is indicated by olive circles+solid line . The black dashed lines represent the bare Landau energy $\hbar \omega _c(N+1/2)$, while the orange and violet dashed lines correspond to the bare excited states $\hbar \omega _{\text {LO}}+(p_1+1/2)\hbar \omega _{c}$ and $\hbar \omega _{A_1}+(p_2+1/2)\hbar \omega _{c}$. Two excitations for $p_i$ ($i=$1,2) are included.

#### PF vs. DP interactions

Figure 2 illustrates the total MP energies as a function of *B* (red solid lines) for $n=2$. The magnetic field is measured in terms of the reduced cyclotron frequency $\omega _c /\omega _{A_1}$. The parameters used for MoS$_2$ and WS$_2$ are listed in Table 1. Plotted are the renormalized polaron ground state level for $N=2$ and the first two excited-state energies (solid lines) for each index $p_i$ ($i=$1,2). The dashed lines represent the unperturbed energy spectrum with the black and orange (violet) color for the Landau level and the excited-state energies related to the phonon frequency $\omega _ {\text {LO}}$ ($\omega _ {A_1}$), respectively. We observe two anticrossings at $\omega _ {c}/\omega _ {A_1} \approx 0.47$ (0.42) and 0.5 for MoS$_2$ (WS$_2$) between the Landau energy $N=2$ and the asymptotes $\hbar \omega _{\text {LO}}+\hbar \omega _{c}/2$ and $\hbar \omega _{A_1}+\hbar \omega _{c}/2$. The same occurs at high magnetic fields when $\omega _ {c}/\omega _ {A_1}\approx$ 0.95 (0.85) and 1.0 due to the crossover with the unperturbed excited state with $p_1=p_2=2$. In addition, using Fig. 2, we compare the relative contribution of the PF interaction (olive circles+solid line) to the total MP energy ${\hat{\epsilon }}_2$. For WS$_2$, the anticrossings are dominated by the PF interaction with a very small contribution of the short-range DP interaction. At the same time, for MoS$_2$ both interactions must be taken into account for an adequate description of the MP spectrum.

**Figure 3:**
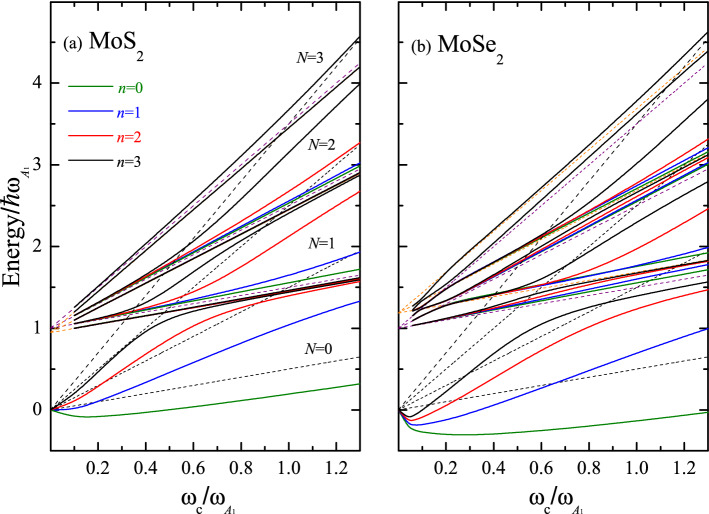
Magneto-polaron spectrum, ${\hat{\epsilon }}_n/\hbar \omega _{A_1}$, as a function of $\omega _c/\omega _{A_1}$ for the set of four states with the quantum numbers $n=$ 0, 1, 2, and 3 for (**a**) ML MoS$_2$ and (**b**) ML MoSe$_2$. The solid lines show the renormalized Landau levels calculated according to Eqs. (6) and (7). The black dashed lines represent the bare Landau level energies with $N=$ 0, 1, 2, and 3, while the orange and violet dashed lines display the bare excited states $\hbar \omega _{\text {LO}}+(p+1/2)\hbar \omega _{c}$ and $\hbar \omega _{A_1}+(p+1/2)\hbar \omega _{c}$  (*p*=0, 1, and 2). For each label *p*, the first four dressed excitations ($n=$ 0, 1, 2, and 3) are included.

**Figure 4:**
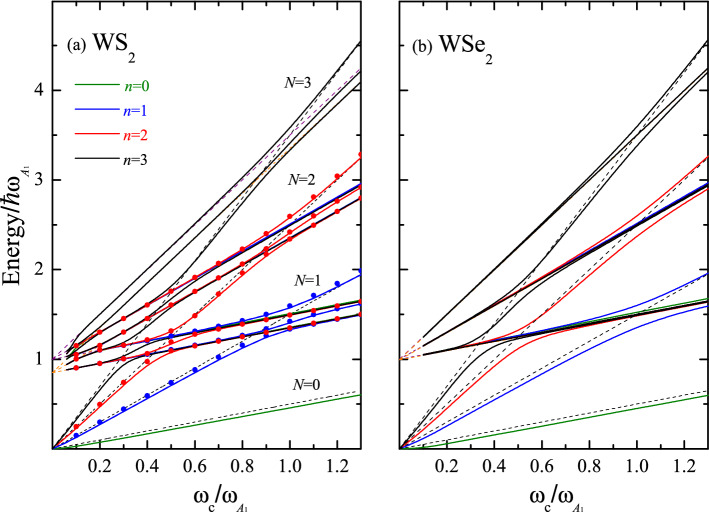
The same as Fig. 3 for (**a**) WS$_2$ and (**b**) WSe$_2$. Dots represent the two-level approximation of Eq. (2).

#### MP spectrum for MoX$_2$ and WX$_2$ (X=S and Se)

Figures. 3 and 4 represent the rich structure of the MP spectrum. The first four states with the quantum numbers $n=$0, 1, 2, and 3 are plotted as a function of $\omega _c/\omega _{A_1}$ for MoX$_2$ and WX$_2$ with (a) X$=$S and (b) X$=$Se. The parameters employed for the calculation of ${\hat{\epsilon }}_n/\hbar \omega _{A_1}$ are provided in Table 1. For a better perception, the energy branches belonging to the various sets with the same quantum numbers $n=$ 0, 1, 2, and 3, are plotted with different colors. In all considered materials for each Landau level *N*, there are three branches involving the phonons $\omega _{\text {LO}}$ and $\omega _{A_1}$ at the cyclotron resonance transition energies as given by Eq. (1). The splitting values obtained for MoS$_2$ and MoSe$_2$ are greater than those for WS$_2$ and WSe$_2$ as a consequence of the fact that the coupling constants ($D_c$ and $\mathbb {G}_{Ph}$ in Table 1) for the series of Mo are larger than those for the series of W. The same holds true for the pair MoS$_2$ and MoSe$_2$, for which the ratio of the PF coupling constants is equal to 0.66, while DP coupling constants have close to each other values. For WS$_2$ and WSe$_2$, there exists a compromise between the both types of the EPI ($\mathbb {G}_{Ph}$ constant is bigger in WS$_2$, than in WSe$_2$ in contradistinction to $D_c$ constant) resulting in the rather similar spectra. The enhancement of the energy gaps observed in WSe$_2$ as compared to WS$_2$ is due to the fact that $\omega _{\text {LO}} \simeq \omega _{A_1}$ leading to a reinforcement of the resonance process. The renormalization of the Landau levels reflects the coupling between the neighboring Landau states via their interaction with the polaron threshold state^8^ and —at high magnetic fields—the fan lines approach the bare Landau energy $\hbar \omega _c(N+1/2)$ or the asymptotes $\hbar \omega _{\text {LO}}(\omega _{A_1})+(p_{1(2)}+1/2)\hbar \omega _{c}$^35^.

In general, the sequence of the MP energies is as follows: 1. The ground-state lines are asymptotic to the line $\omega _{\text {LO}}$ ($\omega _{A_1}$)+$\omega _c/2$ ($\omega _{A_1}$ for WSe$_2$). 2. At $\omega _c^{(1)}=\omega _{\text {LO}} (\omega _{A_1})/N$ for the first crossover, the second renormalized energy is asymptotic to $\omega _{A_1} (\omega _{\text {LO}})+\omega _c/2 (\omega _{\text {LO}}$ for WSe$_2$). 3. If $n>$1, the third levels become the first excited energies of the next anticrossing, showing the ladder-like structure that couples the adjacent Landau states (these characteristics were evaluated for the first time in Ref.^8^ explaining fan plots of resonance energies vs magnetic field obtained from magneto-Raman profiles in InP bulk semiconductor^44^. The coupling between more than two levels becomes necessary, in order to obtain a correct description of the energy spectrum as a function of *B* away from resonances.) These states bend upward close to the second line of the excited state, i.e. $\omega _{\text {LO}}$($\omega _{A_1}$)+5$\omega _c/2$. 4. For the second anticrossing ($\omega _c=\omega _{\text {LO}}(\omega _{A_1})/(N-1))$, the first and second states approach $\omega _{\text {LO}}(\omega _{A_1})+5\omega _c$/2 and $\omega _{A_1} (\omega _{\text {LO}})+5\omega _c/2$, respectively. 5. As the field increases ($\omega _c \rightarrow \infty$), the third anticrossing of a given set *n* approaches the Landau energy $\hbar \omega _c(N+1/2)$.

Importantly, as follows from Figs. 2, 3, and 4, the gaps observed at each crossing point are determined not only by the polar-phonon frequency $\omega _{\text {LO}}$ and the PF coupling constant, but also by the homopolar-phonon frequency $\omega _{A_1}$ and the DP coupling constant. The assumption that the latter parameter is proportional to the PF coupling constant, typical of 3D semiconductors, underestimates the true value of the gap^35^. (In Ref.^35^, incorrect values of the coupling constants $\mathbb {G}_{Ph}$ were used, which is reported in^45^. Corrections to the previously calculated magnitudes for the electron-phonon interaction for the MX$_2$ family were reported in Ref.^46^.) The shift and the slope of the ground-state energy strongly depend on the DP coupling constant. In WS$_2$ and WSe$_2$, the slope of the ground-state energy shows a linear behavior as a function of ${ B}$, and it is possible to define an effective mass $m_{eff}=m_{eff}({ B})$ for the electronic state. This is not the case for MoS$_2$ and MoSe$_2$ MLs, where the interplay between the PF and DP interactions breaks the linear dependence of ${\hat{\epsilon }}_n$ on ${ B}$. If the DP is switched off, the standard dependence of the MP energy on the magnetic field is recovered (compare Figs. 2a and 3b). In general, the inclusion of the short-range $A_1$-homopolar DP interaction gives rise to a different qualitative behavior of the MP effects for the two considered series of MoX$_2$ and WX$_2$ materials. Furthermore, the polaron energy shown in Figs. 3 and 4, which highlight the presence of three excitation branches in the MP spectrum, opens a new perspective for the control of the phenomena related to correlated electronic states. A similar result was reported in Ref.^43^ for experimentally detected Raman spectra in graphene, where the Raman shift as a function of magnetic field showed the existence of three branches of electronic excitations involving the optical phonons at the points K and $\Gamma$ of the BZ.

#### Two-level approximation

A reliable analytical expression considering the two Landau-level coupling with two optical phonons of different symmetries provides a very desirable perspective for the determination of important parameters, such as the anticrossing polaron-energy gap and the strengths of EPI in TMD. In a picture of two Landau levels in coupling with one phonon branch, we have at each crossover point energy splitting in two-branches . Nevertheless, in TMD compounds, the MP resonances are beyond this simple picture. For the *B* values close to the crossing points, as given by Eq. (1), the general expressions Eqs. (6), (16) and (20) (see Sect. **General Formulation**) imply that the set of the renormalized energies ${\hat{\epsilon }}$ are approximated by

2 $$\begin{aligned} {\hat{\epsilon }}_{ap }(N,N^{'})= & {} \hbar \omega _c\left[ N+1/2+ \frac{\alpha _{\text {DP}}\hbar \omega _{A_1}}{{\hat{\epsilon }}_{ap}-\hbar \omega _{A_1}-\hbar \omega _{c}(N^{'}+1/2)} +\frac{\alpha _{\text {PF}}\hbar \omega _{A_1}f_{N^{'},N}(r_0/l_c)}{{\hat{\epsilon }}_{ap}-\hbar \omega _{\text {LO}}-\hbar \omega _{c}(N^{'}+1/2)} \right] , \end{aligned}$$

where the contribution of the imaginary part is assumed zero and $N^{'}=0, 1, 2,...,N-1$. Equation (2) can be represented in terms of a third-order polynomial in ${\hat{\epsilon }}_{ap}$ involving two independent EPIs. From this polynomial equation, three real solutions follow, which are associated to the energy levels at each anticrossing. In Fig. 4a, the approximate energies ${\hat{\epsilon }}_{ap}$ rescaled by the phonon energy $\hbar \omega _{A_1}$ are compared with those obtained by the exact numerical solution of Eqs. (6) and (7). Dots correspond to the three approximate solutions as a function of ${ B}$ and are represented, for the sake of comparison, with the same color as the obtained exact solutions labelled according to the quantum number *n*. From Fig. 4a, it is clear that the approximative solutions given by Eq. (2) are a powerful tool to evaluate the coupling constants $\mathbb {G}_{Ph}$ and $D_c$ from the available experimental magneto-optical measurements. In particular, they will be especially useful for the DP, where experimental evidences are very scarce. Nevertheless, the solutions ${\hat{\epsilon }}_{ap}$ are valid wherever the MP resonances occur, if the involved parameters, $\alpha _{\text {DP}}$ and $\alpha _{\text {PF}}f_{N',N}$, are small enough. For the PF interaction, the function $f_{N',N}$ plays an important role depending on the Landau level *N* under consideration. According to the values reported in Table 1 for MoS$_2$ and MoSe$_2$, there exist differences of the order of 20 % between the results of Eq. (2) and the exact numerical solutions. It is necessary to emphasize that the values of $D_c$ of 5.8 eV/Å and 5.2 eV/Å, as reported in Ref.^40^ for MoS$_2$ and MoSe$_2$, respectively, are not within the range of validity of the approximate Eq. (2). Instead, if $D_c$ are 1.75/Å and 1.10 eV/Å, as reported in Ref.^47^, the two-level approximation of Eq. (2) works very well.

#### Life-time broadening

**Figure 5:**
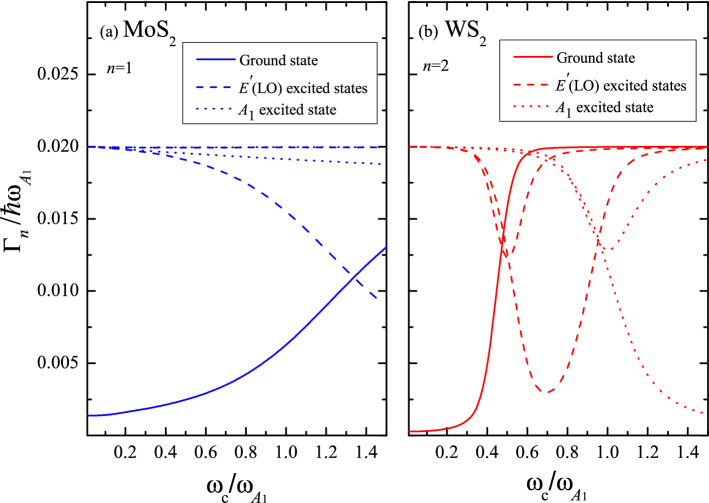
Life-time broadening (in units of $\hbar \omega _{A_1}$) vs the relative cyclotron frequency $\omega _c/\omega _{A_1}$ for the quantum level $n=1$ ($n=2$) in MoS$_2$ (WS$_2$). Solid line corresponds to the ground-state polaron level. Dashed and dotted lines represent the first two excited states for the LO and $A_1$-homopolar modes, respectively.

In the case under consideration, the energy uncertainty is caused by the PF and DP electron-phonon couplings. The imaginary part of the self-energy $\Gamma =\text {Im}[S(E,N)]$ is related to the particle life-time broadening. For a given value of *B*, the solution of Eqs. (6) and (7) provides the set of energies ${\hat{\epsilon }}_{n}$, ordered in Figs. 3 and 4 according to increasing values, and the corresponding parameters $\Gamma _n(B)$. As an illustration, in Fig. 5, the calculated level widths $\Gamma _{n=1}$ and $\Gamma _{n=2}$ as a function of *B* for the perturbed ground-state energy level and the first two excited states are plotted for both involved optical phonons in MoS$_2$ and WS$_2$, respectively. Following the results of Fig. 3 for ${\hat{\epsilon }}_{n=1}$ and Fig. 4 for ${\hat{\epsilon }}_{n=2}$ near the resonances, the width of the ground-state level (solid line) increases, while the broadenings of the excited-states reach their minimum values at the crossing points with $\omega _{\text {LO}}$ (dashed lines) and $\omega _{A_1}$ (dotted lines).

## Conclusions

Magneto-polaron resonances in ML of MoS$_2$, MoSe$_2$, WS$_2$, and WSe$_2$ are studied beyond the two-level approximation. Optical phonons with $E{'}$ and $A_1$-symmetries break the degeneracy between the Landau levels and the excited states $\hbar \omega _{\text {LO}}+(p_1+1/2)\hbar \omega _c$, $\hbar \omega _ {A_1}+(p_2+1/2)\hbar \omega _c$ with the emergence of three resonant branches in the MP contribution at each crossing point. The theoretical model based on the Green’s function formalism captures the new peculiarities of the renormalized energy spectrum: the coupling between the neighboring Landau levels and the life-time broadening as a function of *B*. The ladder-like structure modifies the energy spectrum through resonances and does not remain pinned at the MP threshold (see Figs. 3 and 4).

The energy gaps, associated to the anticrossings, the energy shifts and the effective masses depend on the long-range PF and short-range DP interactions. A generalized two-level model including the contributions of both EPIs, is sufficient to describe the main features of renormalized energies (see Fig. 4a) as long as the transitions between neighboring Landau levels are negligible (weak-coupling limit).

One particularly exciting aspect of the obtained results is their application to the resonant magneto-Raman scattering, where the strong anticrossings and the three excitation branches in Landau-level fan plots can be obtained from magneto-Raman profiles of the LO and $A_1$-homopolar modes scattering intensities. Thus, the present results are a guideline for magneto-optical and cyclotron-resonance measurements, which will provide unique, otherwise unachievable, information about the PF and DP coupling constants in ML of TMDs toward optimization of their diverse functionalities.

Among other topics, the evaluation of the photo-carrier scattering rate under a MP resonance, where the cyclotron frequency is of the order of $\omega _c^{(\text {LO})}(B_{\text {LO}})$ and $\omega _c^ {(A_1)}(B_{A_1})$ offers an attractive research field for finding new peculiarities in hot-magneto-photoluminescence. In addition, the discrete nature of the quantum energy levels described by Eqs. (4) and (5), provides a good platform for analysis of the quantum transport phenomena in an electron gas under resonance conditions by virtue of EPI. Finally, the existence of a flat band in the twisted 2D TMDs^36,48^ allows for the investigation of strongly correlated electronic states, in particular, the influence of the MP resonances regime on filling factors.

The fundamental results of the present paper are: 1. The D$_{3h}$ symmetry of the lattice vibrations of single layer of TMD dictates that the MP spectrum depends on the interplay between two interactions, the long-range (PF) and short-range (DP). 2. New energy branches in the MP spectrum involving the $E^{'}(\text {LO})$ and $A{_1}$ optical phonons are obtained. 3. The shift and the slope of the ground-state energy strongly depend on the PF and DP coupling constants. 4. The renormalized energies vs magnetic field show staircase-like structure reflecting a mixing effect of the bare Landau level systems and the bare excited $E^{'}(\text {LO})$ and $A{_1}$ modes. 5. There are two different sets of cyclotron frequencies as given by Eq. (1). 6. At resonances, the gap widths at the anticrossing points are determined by two contributions, PF and DP interactions with ${\omega _{\text {LO}}}$ and $\omega _{A_1}$ frequencies, which are responsible for the appearance of three-levels branches in the MP spectrum. 7. The key solution of the set of Eqs. (6) and (7) provides the polaron energy and its life-time broadening as a function of *B*. The present method and the obtained results can be extended to other TMD materials.

## Methods

### General formulation

For the description of the MP resonance we employed the Green’s function method that reveals the inherent symmetries of phonons in the TMD and their influence on the MP quasiparticles. Other existing methods (for example, the modified Wigner- Brillouin perturbation theory) allow one to consider the influence of the EPIs in the MP spectrum and the coupling between the Landau states^4^, rather than the direct evaluation of the life-time and the interconnection between these MP characteristics as a function of the external magnetic field.

We consider a 2D TMD in an external uniform magnetic field ${\textbf {B}}=B{\textbf {e}}_z$ applied along the *z*-direction perpendicular to the plane of the sample. For a description of the MP properties, we start from the Dyson equation for the full Green’s function *G*(*E*, *N*)^49^ for a particle in a static *B*

3 $$\begin{aligned} G(E,N) = G^{(0)}(E,N)+ G^{(0)}(E,N)\sum _{M}S(E,N,M)G(E,M)\;, \end{aligned}$$

where $G^{(0)}(E,N)$ is the one-particle unperturbed Green’s function at $T=0$ K, *S*(*E*, *N*, *M*) is the self-energy considering the interaction of the electronic Landau levels with the optical phonons and the sum is taken over all Landau states. Assuming a free 2D electron gas with a parabolic dispersion and an isotropic effective mass *m* under a constant *B* and neglecting the spin degree of freedom, the electronic wave function can be cast as

4 $$\begin{aligned} \psi _{N,k_y}= & {} \frac{1}{\sqrt{L}}e^{ik_{y}y}\varphi _{N}(x-x_{0}(k_{y}))\;;\;\;\;\;\;\; (N=0,1,2,...)\;, \end{aligned}$$

where $k_{y}$ is the particle wave vector, $\varphi _{N}$ the harmonic oscillator functions, $x_0=-k_yl^2_c$ the center of the orbit, $l_c=(\hbar c/eB)^{1/2}$ the Landau magnetic length and *L* the normalization constant^8^. The corresponding energy, without EPI, is $\epsilon _{N}=\hbar \omega _{c}(N+1/2)$ with $\omega _{c}=eB/mc$. In the Landau representation, the zero-temperature bare electron Green’s function is $G^{(0)}(E,N)=[E-\epsilon _{N}-i\delta ]^{-1}$ with $\delta$ a residual life-time broadening^50^. Solving the Eq. (3) for the dressed Green’s function *G*(*E*, *N*), we obtain the resonant MP energy. The contribution of the self-energy to the renormalized Green’s function is determined by the EPIs. In 2D TMDs, at the center of the Brillouin Zone (BZ), there exist in-plane optical phonons (degenerate LO and TO branches) and one out-of-plane mode (ZO-homopolar branch) belonging to the irreducible representations $E^{'}(\text {LO})$ and $A_1$, respectively^51^. The symmetries of these phonons are responsible for the electron intra-valley transitions via the long-range PF interaction for the LO-phonons and the short-range DP mechanism for the $A_1$-homopolar mode. Hence, both interactions have to be summed up for a correct evaluation of the self-energy: $S(E,N,M)=S_{\text {PF}}(E,N,M)+S_{\text {DP}}{(E,N,M})$. It is possible to show that *S*(*E*, *N*, *M*) is diagonal in the Landau quantum numbers *N*, *M*, that is $S(E,N,M)=\delta _{N,M}S(E,N)$ (see below). Thus, the Dyson equation for the full Green’s function *G*(*E*, *N*) is reduced to

5 $$\begin{aligned} G(E,N) = \frac{1}{\left[ G^{(0)}(E,N)\right] ^{-1} - \left[ S_{\text {DP}}(E,N)+S_{\text {PF}}(E,N)\right] }\;. \end{aligned}$$

The complex energy is $E=\hat{\epsilon }+i\Gamma$, where ${\hat{\epsilon }}=$Re[*E*] and $\Gamma =$Im[*E*] are the MP energy and its life-time broadening, respectively. The pole of the dressed Green’s function determines the renormalized energy $\hat{\epsilon }(B)$, while the life-time broadening $\Gamma (B)$ is given by $\text {Im}[G(E,N)^{-1}]$^8,49^. From Eq. (5), we derive

6 $$\begin{aligned} {\hat{\epsilon }} = \hbar \omega _c\left( N +1/2\right) + \text {Re}\left[ S_{\text {DP}}(E,N)+S_{\text {PF}}(E,N) \right] \;, \end{aligned}$$

and

7 $$\begin{aligned} \Gamma = \text {Im}\left[ S_{\text {DP}}(E,N)+S_{\text {PF}}(E,N)\right]. \end{aligned}$$

Equations (6) and (7) are a set of two coupled transcendental non-linear equations. Solving them simultaneously, we obtain in what follows the energy spectrum ${\hat{\epsilon }}(B)$ as well as the the life-time broadening $\Gamma (B)$.

### Self-energy

In 2D TMDs, the LO and $A_1$(ZO)-modes are responsible for the particle intra-valley transitions at the K point. Thus, the long-range PF and short-range DP interactions must be taken into account for a correct evaluation of the self-energy. The general structure of the EPI Hamiltonian is cast as

8 $$\begin{aligned} {\hat{H}}_{j}= \sum _{{\textbf {q}}} \left[ C_{{\textbf {q}}}^{(j)}e^{i\small {{\textbf {q}}\cdot \varvec{\rho }}}{\hat{b}}_{{\textbf {q}}}+C_{\textbf {q}}^{{(j)}\dag }e^{-i\small {{\textbf {q}}\cdot \varvec{\rho }}}{\hat{b}}_{{\textbf {q}}}^\dag \right], \end{aligned}$$

where $C_{{\textbf {q}}}^{(j)}$ is the coupling constant, $j=$ PF and DP, $\varvec{\rho }$ the in-plane coordinate and ${\hat{b}}_{{\textbf {q}}}$ (${\hat{b}}_{{\textbf {q}}}^\dag$) the annihilation (creation) phonon operator with the phonon wave vector ${\textbf {q}}$. Employing Eqs. (4) and (8), the polaron effect is considered through the self-energy *S*(*E*, *N*, *M*) of the Green’s function of Eq. (5). Considering the electronic wave function of Eq. (4), the irreducible self-energy (obtained when keeping only the Feynman diagrams, which cannot be separated into two disconnected pieces^49,52^) to the lowest order in the coupling constant $C_{{\textbf {q}}}^{(j)}$ can be written as

9 $$\begin{aligned} S_{j}(E,N,M)= & {} \sum _{N',k^{\prime }_y,k^{\prime \prime }_y,{\textbf {q}}}G^{(0)}(E-\hbar \omega _0,N') C_q^{(j)} \langle N',k^\prime _{y}\vert e^{i\small {{\textbf {q}}\cdot \varvec{\rho }}}\vert N,k_{y}\rangle \arrowvert C_q^{*(j)}\langle M,k_{y}^{\prime \prime }\vert e^{i\small {{\textbf {q}}\cdot \varvec{\rho }}}\vert N',k_{y}^{\prime }\rangle \end{aligned}$$

10 $$\begin{aligned}= & {} \sum _{N',k^{\prime }_y,k^{\prime \prime }_y,{\textbf {q}}} G^{(0)}(E-\hbar \omega _0,N') \arrowvert C_q^{(j)}\arrowvert ^2\mathscr {L}_{N',N}({\textbf {q}})\delta _{k^{\prime }_{y},k_{y}-q_{y}} \mathscr {L}^{*}_{M,N'}({\textbf {q}})\delta _{k^{\prime \prime }_{y},k_{y}^{\prime }-q_{y}}. \end{aligned}$$

The matrix element $\mathscr {L}_{N',N}({\textbf {q}})$ is

11 $$\begin{aligned} \mathscr {L}_{N',N}({\textbf {q}})=\int _{-\infty }^{\infty }\varphi _{N'}(x-x_0(k^{\prime }_{y}) )\varphi _N(x-x_0(k_{y}))e^{-iq_{x}x} dx, \end{aligned}$$

and $k^{\prime }_{y}=k_{y}-q_y$. Integrating over the coordinate *x*, we obtain

12 $$\begin{aligned} \mathscr {L}_{N',N}({\textbf {q}})= & {} e^{-\frac{i}{2} q_x(k_y+k^{\prime }_y)l_c^{2}}e^{-\frac{1}{4}l_c^2q_{\perp }^2}\times \nonumber \\{} & {} \left\{ \begin{array}{lcl} 2^{\frac{N-N'}{2}}\sqrt{\frac{N'!}{N!}}\left[ \frac{l_c}{2}(q_y-iq_x)\right] ^{N-N'} L_{N'}^{N-N'}\left( \frac{1}{2}l_c^2 q_{\perp }^2\right) \,, &{}\text {for}\,&{} N \geqslant N',\\ 2^{\frac{N'-N}{2}}\sqrt{\frac{N!}{N'!}}\left[ -\frac{l_c}{2}(q_y+iq_x)\right] ^{N'-N} L_{N}^{N'-N}\left( \frac{1}{2}l_c^2 q_{\perp }^2\right) \,, &{} \text {for} &{} N<N', \end{array} \right. \end{aligned}$$

with $L_{p}^{q}(z)$ the generalized Laguerre polynomials^53^. Representing *q* in the cylindrical coordinates and integrating over the polar angle result in $S_{j}(E,N,M)=\delta _{N,M}S_{j}(E,N)$, where

13 $$\begin{aligned} S_{j}(E,N)&=l_c^{-2}&\sum _{N'} G^{(0)}(E-\hbar \omega _{0},N') \frac{S}{2\pi }\int _0^{\infty }\arrowvert C_Q^{(j)} \arrowvert ^2T_{N',N}(Q)dQ, \end{aligned}$$

and

14 $$\begin{aligned} T_{N',N}(Q)=e^{-Q}{} & {} \left\{ \begin{array}{lcl} \frac{N'!}{N!}Q^{N-N'}\arrowvert L_{N'}^{N-N'}(Q)\arrowvert ^2 &{} \text {for}\,, &{} N \geqslant N'\;, \\ \frac{N!}{N'!}Q^{N'-N}\arrowvert L_{N}^{N'-N}(Q)\arrowvert ^2 &{} \text {for}\,, &{} N<N'. \end{array} \right. \end{aligned}$$

**Table 1:** Employed parameters for the calculation of the energy spectrum from the set of Eqs. (6) and (7). The value of $\delta /\hbar \omega _{A_1}=0.02$ is used. *a* is the lattice constant and $\mathbb {G}_{Ph}$ is the PF coupling constant of Eq. (19).

	$\hbox {MoS}_{2}$	$\hbox {MoSe}_{2}$	$\hbox {WS}_{2}$	$\hbox {WSe}_{2}$
$m/m_0$^54^	0.43	0.49	0.35	0.39
$\hbar \omega _{A_1}$ (meV)^41^	49.5	29.4	51	30.2
$\hbar \omega _{\text {LO}}$ (meV)^41^	46.9	34.9	43.3	30
$D_c$ (eV/Å) $^{\textrm{a}}$	5.8, 1.75	5.2, 1.10	3.1	2.3
$\mathbb {G}_{Ph}$ (eV)^41^	0.356	0.538	0.162	0.301
*a* (Å)^41^	3.1635	3.2974	3.1627	3.2954
$r_0$ (Å)^41^	46.018	53.3517	41.898	48.704

$^{\textrm{a}}$ The highest $D_c$ values of 5.8 eV/Å and 5.2 eV/Å for MoS$_2$ and MoSe$_2$, respectively, were obtained adjusting the ab initio calculations with the relaxation time^40^. The values of 1.75 eV/Å and 1.10 eV/Å were derived by employing the pseudopotential method^47^. This large variation in values for $D_c$ makes a marked difference in the resulting MP spectrum, in particular, in the relative contribution of the PF and DP interactions to ${\hat{\epsilon }}$ and $\Gamma$. In the present calculations, the values of 5.8 eV/Å and 5.2 eV/Å are used.

#### Intra-band deformation potential

The DP characterizes the changes of the band energy under mechanical deformations of the primitive unit cell due to the optical lattice oscillations of the out-of-plane $A_1$-homopolar branch. In first-order approximation, the EPI is independent of the phonon wave vector, and the DP coupling constant is^41^

15 $$\begin{aligned} C_q^{\text {DP}} = \left( \frac{\hbar }{2\rho _mA N_c\omega _{A_1}}\right) ^{1/2}D_c, \end{aligned}$$

where $\rho _m$ is the 2D reduced mass density associated with the two chalcogen atoms, $\omega _{A_1}$ the ZO-phonon frequency, $D_c$ the deformation potential constant, $A=\sqrt{3}a^2/2$ the area of the unit cell, *a* the lattice constant, and $N_c$ the number of cells. Taking advantage of the fact that $C_q^{\text {DP}}$ is independent of the phonon wave vector and employing the result $\int _0^{\infty }T_{N^{'},N}(Q)dQ=1$^55^, the self-energy acquires the form

16 $$\begin{aligned} S_{\text {DP}}(E,N) = \hbar \omega _{c}\hbar \omega _{A_1}\alpha _{\text {DP}}&\sum \limits _{N'}[E-\hbar \omega _{A_1}-\hbar \omega _{c}(N'+1/2)-i\delta ]^{-1}\; \end{aligned}$$

with

17 $$\begin{aligned} \alpha _{\text {DP}}=\frac{m}{4\pi \rho _m }\left( \frac{D_c}{\hbar \omega _{A_1}}\right) ^2\;. \end{aligned}$$

#### Pekar–Fröhlich interaction

The in-plane motion of the positive M-ion relative to the X$_{i}$-ions is responsible for the long-range interaction. The macroscopic electrostatic potential associated with the in-plane LO-vibrations acts on the electron, leading to the EPI valid for a ML of TMD with a coupling constant^41^

18 $$\begin{aligned} C_q^{\text {PF}}= & {} \frac{\mathbb {G}_{Ph}}{\sqrt{N{_c}}(1+r_0q)}, \end{aligned}$$

19 $$\begin{aligned} \mathbb {G}_{Ph}= & {} \left( \frac{2 \pi ^2 e^2\hbar \alpha ^2}{\rho _mA\omega _{\text {LO}}}\right) ^{1/2}, \end{aligned}$$

where $\omega _{\text {LO}}$ is the in-plane phonon-frequency at ${q}={0}$, $\rho _{m}$ the mass density with the reduced atomic mass $\mu =m_{_{\text {M}}}^{-1}+(2m_{_{\text {X}}})^{-1}$, $\alpha$ the coupling constant between the atomic displacement and the in-plane macroscopic electric field, and $r_0$ the screening parameter^41^. In this case, the contribution of the long-range interaction to the self-energy is

20 $$\begin{aligned} S_{\text {PF}}(E,N)=\hbar \omega _{c}\hbar \omega _{A_1}\alpha _{\text {PF}}\sum _{N'}\frac{f_{N',N}(r_0/l_c)}{E-\hbar \omega _{\text {LO}}-\hbar \omega _c\left( N'+\frac{1}{2}\right) -i\delta }, \end{aligned}$$

where

21 $$\begin{aligned} \alpha _{\text {PF}}=\frac{A\mathbb {G}_{Ph}^2}{2\pi \hbar \omega _{A_1}}\frac{m}{\hbar ^2} \end{aligned}$$

and

$$\begin{aligned} f_{N',N}(z)=\int \limits _0^\infty \frac{dQ}{\left( 1+z\sqrt{2Q}\right) ^2} T_{N',N}(Q). \end{aligned}$$

From the self-energy as given by Eqs. (16) and (20), it follows immediately that a mixing effect for the MP energy is present. The summation over all Landau levels leads to a coupling between different states. For a given quantum number *N*, the relative contribution of the other states $N'$ to *E* depends on the coupling constants $\alpha _{\text {DP}}$ and $\alpha _{\text {PF}}$.

## Data Availability

The datasets used and/or analysed during the current study are available from the corresponding author on reasonable request.
